# Ethnicity and Socioeconomic Disparities in Clinical Trial Participation for Ovarian Cancer: A Retrospective Observational Study in London

**DOI:** 10.3390/cancers16213590

**Published:** 2024-10-24

**Authors:** Karim H. El-Shakankery, Joanna Kefas, Kieran Palmer, Andrew Houston, Uma Mukherjee, Kangbo Gao, Weiteen Tan, Shanthini M. Crusz, Michael J. Flynn, Jonathan A. Ledermann, Michelle Lockley, Mary McCormack, Nicola MacDonald, Shibani Nicum, Michael John Devlin, Rowan E. Miller

**Affiliations:** 1 Department of Medical Oncology, University College London Hospital, London NW1 2BU, UK; 2 St Bartholomew’s Hospital, Barts Health NHS Trust, London EC1A 7BE, UK; 3 Barts Life Sciences, Barts Health NHS Trust, London EC1A 7BE, UK; 4 Digital Environment Research Institute, Queen Mary University of London, Charterhouse Square, London EC1M 6BQ, UK; 5 Barts and The London School of Medicine and Dentistry, Garrod Building, Turner Street, Whitechapel, London E1 2AD, UK; 6 UCL Cancer Institute, University College London, Huntley Street, London WC1E 6DD, UK; 7 Centre for Cancer Genomic and Computational Biology, Barts Cancer Institute, Queen Mary University of London, Charterhouse Square, London EC1M 6BQ, UK

**Keywords:** ethnicity, socioeconomic status, health outcomes, cancer mortality, health inequalities, clinical trials

## Abstract

Background: Ethnic and socioeconomic disparities in cancer outcomes are exacerbated by clinical trial underrepresentation. This study aims to identify inequalities in ethnicity and socioeconomic features among ovarian cancer clinical trial participants in two London cancer centres. Methods: All ovarian cancer patients treated between 2017 and 2022 were included. Patients participating in clinical trials were classified as the trial population (TP); the remainder were considered the non-trial population (NTP). Data on disease characteristics and sociodemographic features, including ethnicity and Indices of Multiple Deprivation (IMD) deciles, were accessed from electronic patient records. Results: Of the 892 patients, 212 (24%) were enrolled in trials: 87 in Phase II, 103 in Phase III, and 21 in prospective, non-investigational medicinal product trials. The TP were more likely to be of White ethnicity (72.6% vs. 57.5%; *p* < 0.001), younger (mean age 58 vs. 60; *p* = 0.003), living in less deprived areas (most deprived tercile: 21.2% vs. 34.0%; *p* = 0.004), and English-speaking (95.8% vs. 90.9%; *p* = 0.041). In the multivariate analysis, White ethnicity (*p* < 0.0001), age (*p* = 0.003), IMD decile (*p* = 0.007), and interpreter requirement (*p* = 0.037) were independent predictors of trial participation. Conclusions: Ethnic and socioeconomic inequalities affect trial participation, potentially worsening health disparities in ovarian cancer patients. Strategies to overcome trial recruitment barriers for underserved groups are needed to improve the equity of care.

## Simple Summary

This study on ovarian cancer clinical trials reveals significant underrepresentation of ethnic minorities and socioeconomically deprived individuals compared to the general patient population. Trial participants were predominantly White, English-speaking, and from less deprived areas. Factors such as age, socioeconomic status, and language proficiency were identified as key predictors of trial participation. These findings highlight a critical gap in ensuring diverse representation in clinical research, which is essential for enhancing the applicability of treatment outcomes across different patient demographics. Addressing these disparities is crucial for promoting equity in healthcare access and improving overall cancer care outcomes. Future efforts should focus on implementing inclusive recruitment strategies and collecting comprehensive demographic data to better understand and address barriers to participation among underrepresented groups.

## 1. Introduction

Ethnicity and socioeconomic status (SES) are known determinants of health outcomes and cancer mortality [1,2,3]. In the United Kingdom (UK), health inequalities are worsening despite equal access to healthcare [4,5]. These disparities are often perpetuated by an overemphasis on biological and geographical differences, neglecting the complex intersectionality of individual and organisational factors [6]. Within cancer care, disparities are evident in screening and prevention strategies, the stage at diagnosis, treatment outcomes, clinical trial participation, and survival outcomes [7,8,9]. Clinical research trials are essential for improving cancer outcomes by developing and establishing innovative prevention, diagnostic, and treatment strategies. Since the establishment of the National Cancer Research Network (NCRN), the UK has achieved a world-leading annual clinical trial recruitment rate of 17% [10]. However, addressing disparities in trial participation has so far received less attention and funding. The true extent of the issue is unknown, as data collection on ethnicity and SES is inadequate, even within the NCRN and national cancer registries. Similarly, in the United States, 37% of the 230 trials leading to drug approval between 2008 and 2018 did not report data on participants’ ethnicity [11]. The underrepresentation of minority ethnic groups and socioeconomically deprived communities in clinical trials remains a key issue, impacting treatment opportunities and the applicability of study results to diverse populations [9,12,13].

Ovarian cancer, the eighth most common cancer in women globally, has only a 50% 5-year survival rate, with its incidence predicted to rise by 55% by 2050 [14]. Existing inequities in ovarian cancer outcomes are linked to disparities in the quality of care and overall survival based on ethnicity, insurance coverage, economic stability, and geographical location [15,16]. However, research specifically addressing the diversity of participants in ovarian cancer clinical trials is limited.

University College London Hospitals (UCLH) and St Bartholomew’s Hospital (SBH) are two tertiary cancer centres covering a combined population of 4.3 million people. These centres serve some of the most ethnically diverse, but also socially deprived, areas in London [17]. This study examines differences in ethnicity, socioeconomic deprivation, and other sociodemographic characteristics between trial and non-trial patients to improve our understanding of existing disparities. The findings may inform strategies for clinicians, researchers, and trial sponsors to monitor inequities and develop contextualised solutions to broaden clinical trial diversity, promote equity in ovarian cancer care, and improve overall survival.

## 2. Materials and Methods

### 2.1. Overview

We conducted a multicentre, retrospective observational study to assess if the ovarian cancer trial population (TP) is representative of the general non-trial population (NTP) receiving standard-of-care cancer treatments in two London tertiary cancer centres. The study was conducted and approved as an audit at UCLH and SBH. Patients treated between 2017 and 2022 were included. The cancer subtype clinical codes used for inclusion were “ovarian cancer”, “ovarian carcinoma”, “ovarian neoplasm”, “epithelial ovarian cancer”, “metastatic ovarian cancer”, “metastatic epithelial ovarian cancer”, “high-grade serous ovarian cancer”, “metastatic high-grade serous ovarian cancer”, “low-grade serous ovarian cancer”, “metastatic low-grade serous ovarian cancer”, “high-grade serous fallopian tube”, “endometroid ovarian cancer”, “mucinous ovarian cancer”, and “clear-cell ovarian cancer”. Patients enrolled with registered trial participant identification numbers in phase II, phase III, and prospective non-investigational medicinal product (IMP) trials were considered.

### 2.2. Method

Retrospective data were extracted from electronic medical records (EMRs) and electronic prescribing and medicines administration (ePMA) systems. The data were manually screened for accuracy. Variables collected included the age at diagnosis, ethnicity, primary language, English fluency, need for an interpreter, occupation, registered home address, distance from home to treating hospital, cancer stage, histological subtype, and Eastern Cooperative Oncology Group (ECOG) performance status. Additional data for trial participants included the referring hospital, distance from home to trial centre, trial phase, study sponsor, and duration of trial treatment. Missing data were considered in the analysis without imputation.

Ethnicity was categorised using NHS clinical coding based on the UK 2021 Census as follows: Asian or Asian British; Black (African, Black British, Black Caribbean, Black Other); Other Ethnic Group; Mixed/Multiple Ethnic Groups; White (British, Irish, Other); and Unclassified. For statistical power, these were further grouped into Asian; Black; White; and Other.

Socioeconomic deprivation was determined using the UK Indices of Multiple Deprivation (IMD), which measures relative deprivation based on income, employment, education, health, crime, housing, and living environment. Each patient was assigned an IMD decile based on their home address postcode, ranging from most (Decile One; D1) to least deprived (Decile Ten; D10). The standardised UK IMD calculator was used: https://imd-by-postcode.opendatacommunities.org/imd/2019 (accessed on 18 January 2024). IMD deciles were grouped into three tertiles for data analysis as follows: IMD tertile 0 (most deprived) = IMD 1–3; IMD tertile 1 = IMD 4–7; and IMD tertile 2 (least deprived) = IMD 8–10.

### 2.3. Statistical Analyses

Statistical significance was set at *p* < 0.05. Differences in continuous data between the TP and NTP groups were assessed using the Mann–Whitney test. Differences in categorical data were evaluated with the Pearson’s Chi-Square test. An assessment of co-linearity was carried out using Spearman’s rank correlation. A pair of predictors was deemed to be co-linear should the correlation co-efficient exceed 0.5. Binary logistic regressions were applied for both the univariate and multivariate analyses, considering the variables of ethnicity, IMD tertile, employment status, English language proficiency, and need for an interpreter. Significant variables from the univariate analysis were included in the multivariate analysis, accounting for co-linearity by including only the variable with the strongest association of the co-linear pair. Given the number of variables and dataset size, this strategy aimed to mitigate overfitting concerns associated with more complex models. Statistical analyses were performed using Python version 3.9 programming software.

### 2.4. Ethics

This service evaluation was registered as an audit and approved by University College London Hospitals Trust and Barts Health NHS Trust (SBH reference code 13754). Only retrospective data collected as part of routine standard care were included. As per local guidelines, research ethics council approval or informed patient consent was not required.

## 3. Results

### 3.1. Patient Characteristics

Between 2017 and 2022, a total of 892 patients across both centres received treatment for ovarian cancer (Table 1). Overall, the median age was 61 years (IQR: 51–71). At diagnosis, 72.5% of patients were stage III or IV (using The International Federation of Gynaecology and Obstetrics [FIGO] staging), and 85.4% had an Eastern Cooperative Oncology Group (ECOG) performance status of 0 or 1. Of these patients, 783 (87.8%) had available data on ethnicity; of these, 13.9% were Asian or Asian British, 10.1% were Black (including Black British, Black Welsh, Caribbean, or African), 6.4% were Mixed or Other Ethnic Group, and 69.6% were White. Patients from the most deprived tertile of the IMD constituted 40.1% of the total cohort. Most patients were English-speaking (92.0%), with 7.5% requiring an interpreter during their consultations. Whilst there was a considerable proportion of missing employment status data, 58.1% were employed at the time of diagnosis.

There were 212 (24%) patients enrolled in a clinical trial: 87 (10%) in phase II, 103 (12%) in phase III, and 21 (2%) in prospective non-IMP trials. Trial patients were significantly younger than non-trial patients (mean age 58 vs. 60; *p* = 0.003), had a better performance status (0/1 90.6% vs. 83.8%; *p* < 0.001), lived in less deprived areas (most deprived tercile: 21.2% vs. 34.0%; *p* = 0.004), and were more likely to be of White ethnicity (72.6% vs. 57.5%; *p* < 0.001). Additionally, trial participants were predominantly English-speaking (95.8% vs. 90.9%; *p* = 0.041) and less frequently required an interpreter (2.8% vs. 9.0%; *p* = 0.005).

### 3.2. Univariate Analysis

**Figure 1:**
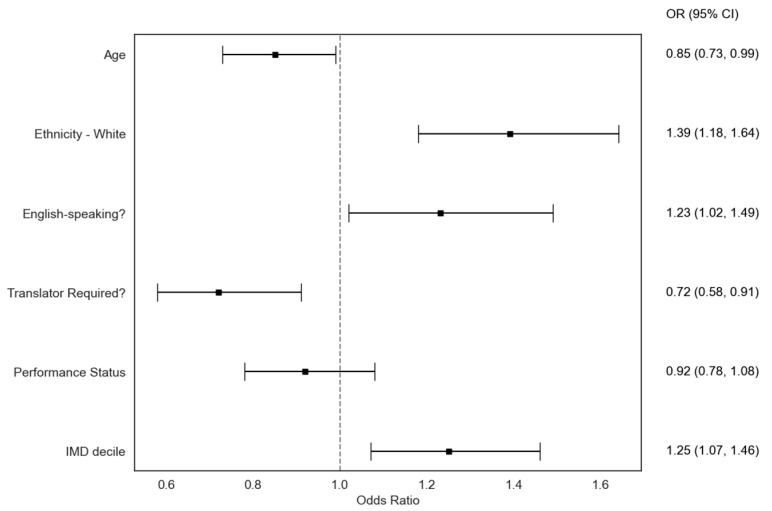
Forest plot demonstrating the independent associations of each predictor with trial participation. Age is treated as a continuous variable, with the odds ratio reflecting the increasing likelihood of trial participation with every increasing year of age. The IMD tertile is treated as ordinal, and the remaining variables are treated as binary.

The univariate analysis revealed that White ethnicity (odds ratio [OR]: 1.39; 95% confidence interval [95% CI]: 1.18 to 1.64), speaking English (OR: 1.23; 95% CI: 1.02 to 1.49), and higher IMD rank were positively associated with clinical trial participation (Table 2). Conversely, older age (OR: 0.85; 95% CI: 0.73 to 0.95) and requiring an interpreter (OR: 0.72; 95% CI: 0.58 to 0.91) were negatively associated with trial recruitment (Figure 1).

### 3.3. Multivariate Analysis

The multivariate analysis included age, IMD decile, interpreter requirement, and ethnicity (Table 3). English language was excluded due to its high correlation index with interpreter requirement (correlation coefficient 0.96) and its lower association with trial enrolment in the univariate analysis. Following multivariate adjustment, age *(p* = 0.003), IMD decile (*p* = 0.007), the requirement for an interpreter (*p* = 0.037), and being of White ethnicity (*p* < 0.0001) remained independent predictors of clinical trial participation.

## 4. Discussion

This study found significant disparities in clinical trial participation among ovarian cancer patients for those of minority ethnicity or residing in more socioeconomically deprived areas. Compared to the general ovarian cancer population, trial participants were more likely to be of White ethnicity, English-speaking, and from a higher IMD rank (less deprived areas). Pertinently, 72.6% of clinical trial patients were of White ethnicity; this must be taken in the context of the higher rate of missing ethnicity data in the non-trial population (13.09% vs. 9.43%; Table 1). Multivariate analysis confirmed that White ethnicity (*p* < 0.0001), age (*p* = 0.003), IMD decile (*p* = 0.007), and the need for an interpreter (*p* = 0.037) were independent predictors of clinical trial participation. These findings highlight that ovarian cancer trial populations do not adequately reflect the broader patient population, leaving those of ethnic minorities or living in more deprived areas underrepresented. The root causes of the observed disparities in trial participation are beyond the scope of this study. While unique factors may exist for the ovarian cancer population, our findings reflect broader barriers to participation in cancer research. We consider our measures of self-reported ethnicity and socioeconomic status to be proxies for broader determinants of health including biology, genetics, comorbidities, employment, housing, education, religion, and experiences of discrimination and mistrust. Therefore, there is no single solution. Addressing these disparities requires collaborative, interdisciplinary approaches that target systemic, clinician-focused, and patient-focused barriers. Clinicians and organisations must tackle workforce diversity, research funding priorities, trial eligibility criteria, and clinician biases. At the patient level, strategies should aim to reduce comorbidities, engage community support systems, improve literacy and language accessibility, build trust, and account for cultural differences in decision-making. Prioritising equity will benefit all patients, and an important area of future research will be evaluating the impact of equity-focused strategies and interventions on health outcomes.

To our knowledge, this is the first UK study to examine clinical trial diversity in ovarian cancer. Historically, clinical trial participation rates for cancer patients are below 5% [18]. A US retrospective cohort study of over 7000 patients with ovarian cancer across 800 sites also reported a 5% trial participation rate [19]. Despite initiatives pushing the UK trial recruitment rate to 17%, there has been an almost 60% decline post-COVID-19 [10,20]. Our study’s higher participation rate of 24% is likely due to the sites involved being specialised tertiary cancer centres with established trial units and active research teams. However, the inclusion of ethnic minorities in clinical trials remains significantly low. The same US cohort study showed that Hispanic and Latino patients were 71% less likely to participate in trials, and those with federal health insurance were 51% less likely compared to those with private insurance [19]. Another US-based, retrospective multicentre study of over 500,000 women with gynaecological cancers found less than 1% trial participation overall, with disproportionately lower enrolment rates for Asian, Black, and Hispanic women [21]. Previous research also shows that White women dominate Gynaecologic Oncology Group (GOG) trials, comprising 83% of participants over nearly 30 years [22]. Initiatives like the National Institute of Health Research (NIHR) INCLUDE project aim to address barriers by defining “underserved” groups and developing strategies to improve their participation in medical research [23]. The development of the “INCLUDE Roadmap” enables trial teams and stakeholders to ask pertinent questions at multiple stages in trial development and set-up. A similar framework specifically for ethnicity has also been developed [24]. Patients of Black ethnicity and those from the most deprived areas face higher mortality rates and are less likely to receive guideline-adherent treatment, even after adjusting for factors such as stage, comorbidity, and performance status [23,24,25]. Although not explored in our study, biological and genomic differences will contribute to differing outcomes amongst ethnic groups, such as BRCA mutations in Ashkenazi Jewish populations [26,27]. However, differences in social, economic, and cultural circumstances may be equally consequential to morbidity and survival.

Our study also identified socioeconomic deprivation and older age as negative predictors of trial participation. Higher socioeconomic levels correlate with increased enrolment based on factors of income, education level, and employment [25]. We measured socioeconomic deprivation using the IMD, which is a composite geographical area-level scoring and in keeping with the majority of existing research. However, this neglects smaller geographical areas and individual-level factors which are more indicative of true socioeconomic status, such as employment status and household income. Ingleby et al. [26] investigated the influence of individual factors such as income, educational attainment, and occupation on cancer survival. They found that although both broadly correlated and contributed to observed inequalities, individual-level inequalities were more significant than area-level effects.

Older cancer patients are often underrepresented in clinical trials due to perceived ineligibility or unsuitability based on comorbidities and functional status. Our study included only one trial participant over 80 years old, compared to 71 non-trial patients in this same age group. The median age at diagnosis of ovarian cancer is 63, but 25% of women diagnosed will be over the age of 74, and 8% will be over the age of 84 [27]. The disproportionately lower recruitment of older patients to clinical trials suggests clinician reluctance and enrolment bias. A refuted barrier is an unwillingness for older patients to participate, as published data suggest that they are equally willing [28,29]. Their involvement is valuable in understanding influences on pharmacological properties that may then impact drug efficacy and safety when used in the older population.

In our study, we sought to record travel distance, recognising that in socioeconomically deprived areas, limited expendable resources make any travel burden a more significant and relevant barrier to clinical trial participation. However, we later excluded this parameter, because the only patients outside the catchment area who received treatment at our study sites were those enrolled in specific trials. Typically, trial participants tend to travel further to reach trial centres, which are often located in affluent, high-population-density areas [30,31]. Although it is reasonable to infer that similar disparities exist in lower-population-density areas, mainly due to financial burdens and geographic variations in ethnicity and socioeconomic deprivation, these factors likely influence both physician-level and patient-level decision-making and motivations for clinical trial referral. More research is needed in low-density areas to better understand geographical inclusion differences.

There are limited published data on patient inclusion in cancer trials for other areas of London and across the UK. A retrospective analysis of 430 patients accessing early-phase trials in South East London similarly found that referrals were less likely for patients from more deprived areas [32]. The observed differences in recruitment of “non-White” ethnicities were lost after adjusting for age, sex, cancer type, and deprivation index. In comparison to other areas of London, including our study catchment area of North London, South London has one of the smallest proportions of people from Black and minority ethnic backgrounds [33]. Another UK-based retrospective cohort study involving 1243 patients in Northern England also identified social deprivation and travel distance as factors affecting referrals and recruitment for early-phase trials [34]. Northern England includes districts with the highest concentrations of neighbourhoods that are amongst the most deprived in England [35]. Although more than 30% of patients in this study had no recorded ethnicity, their 59.6% majority of White British participants were considered reflective of Northen England’s demographic, where over 90% of the population are White British, compared to 36.8% in London [36]. We also explored whether any between-hospital geographical differences existed within our study (Supplementary Figure S1). Whilst there were some differences in baseline characteristics, the univariate analyses assessing trial participation predictors at each site are largely comparable; only the performance status showed a significant inter-hospital difference. This suggests that the results may be applicable across different areas of London. Such results must also be taken in the context of smaller sample sizes, which is a result of hospital site splitting.

A limitation of our study was the lack of reliable high-quality data on individual ethnicity, language, and demographics. Ethnicity was often not reported or not specific enough to allow for appropriate categorisation. Employment status was poorly recorded, and again, when available, it was a limited description of functional capabilities rather than encompassing financial security and educational attainment. Furthermore, the authors acknowledge the complexity of the topic area and the limitations of using univariate and multivariate analyses; however, we also explored using a machine-learning XGBoost modelling approach (Supplementary Methods and Figure S2), which demonstrated largely consistent results and supports the robustness of our conclusions. Another limitation is defining socioeconomic deprivation with biased area-level scores, such as the IMD, which do not capture individual circumstances.

Finally, due to a lack of data, we cannot account for eligibility differences driving clinical trial participation disparities in this study. As a result, the NTP group may include patients who either did not meet eligibility suitability or were suitable for trials but chose to decline participation. Thus, eligibility rates and patient interest were not included in our comparative analysis. However, the authors also highlight that overly restrictive or subjective eligibility criteria can exacerbate disparities by excluding individuals based on factors such as comorbidities, age limits, the language proficiency required for consent, or a lower prevalence of specific biomarkers in certain populations [37]. These exclusions would disproportionately affect underserved groups, contributing to underrepresentation in clinical trials.

Acknowledging these limitations is useful for future studies to better characterise and form targeted solutions to improve clinical trial participation diversity.

## 5. Impact Statement

Improving the representation of trial populations is imperative for several reasons. Firstly, it enhances the generalisability of trial findings, ensuring that research outcomes are applicable across diverse real-world settings. The varied socioeconomic and ethnicity-based lived experiences of participants influence aspects of the cancer experience such as access to care, treatment adherence, and treatment outcomes. Without adequate representation, important differences across diverse populations may go unnoticed. Therefore, deliberate efforts to achieve balanced sociodemographic inclusion are essential for understanding how ethnicity and SES impact the effectiveness and safety of interventions. Ethical standards and regulatory requirements should require evidence that treatments are safe and effective across diverse populations.

Equitable distribution of healthcare innovations requires inclusive trial participation. This approach also provides insights into real-world challenges such as transportation, work commitments, childcare support, and financial constraints that may affect treatment adherence. Policymakers can use this information to allocate resources and support effectively to meet the specific needs of underrepresented groups. Solutions include leveraging high-quality data to measure associations and monitor changes effectively, collaborating closely with affected communities, and designing eligibility criteria that facilitate and ensure the participation of minority groups.

## 6. Conclusions

Those from minority ethnic backgrounds or residing in socioeconomically deprived areas were notably underrepresented in our ovarian cancer clinical trials. For clinical trials to truly reflect the populations they aim to serve, diverse representation is essential. Failing to achieve this not only limits treatment opportunities for individuals from minority groups but also reduces the real-world relevance of study findings. The lack of robust, accurate, and comprehensive ethnicity and sociodemographic data for analyses underscores the need for multisectoral solutions to address the complex intersection of ethnicity, socioeconomics, and cancer outcomes.

## Figures and Tables

**Table 1:** Summary of characteristics of overall, trial, and non-trial populations.

**Continuous Features:**								
**Feature**	**Trial Mean ± SD**	**Trial Median (IQR)**		**Non-Trial Mean ± SD**		**Non-Trial Median (IQR)**		***p*-Value**
Age	57.98 ± 11.8	59.0 (51.0–72.0)		60.46 ± 16.06		63.0 (51.0–72.0)		0.003
**Categorical Features:**								
**Feature**	**Sub-Group**	**All N = 892 (%)**	**Trial N = 212 (%)**		**Non-Trial N = 680 (%)**		**Trial: All**	***p*** **-Value**
Ethnicity	White	545 (61.1)	154 (72.6)		391 (57.5)		0.28	0.003
	Asian	109 (12.2)	17 (8.0)		92 (13.5)		0.16	
	Black	79 (8.9)	11 (5.2)		68 (10.0)		0.14	
	Other	50 (5.6)	10 (4.7)		40 (5.9)		0.2	
	Missing	109 (12.2)	20 (9.4)		89 (13.1)		0.18	
English-Speaking?	Yes	821 (92.0)	203 (95.8)		618 (90.9)		0.25	0.041
	No	69 (7.7)	9 (4.2)		60 (8.8)		0.13	
	Missing	2 (0.2)	0 (0.0)		2 (0.3)		0	
Translator Required?	Yes	67 (7.5)	6 (2.8)		61 (9.0)		0.09	0.005
	No	822 (92.2)	206 (97.2)		616 (90.6)		0.25	
	Missing	3 (0.3)	0 (0.0)		3 (0.4)		0	
Employed	Yes	519 (58.2)	127 (59.9)		392 (57.6)		0.24	0.022
	No	70 (7.8)	8 (3.8)		62 (9.1)		0.11	
	Missing	303 (34.0)	77 (36.3)		226 (33.2)		0.25	
FIGO Stage	1	138 (15.5)	27 (12.7)		111 (16.3)		0.20	0.007
	2	41 (4.6)	12 (5.7)		29 (4.3)		0.29	
	3	329 (36.9)	94 (44.3)		235 (34.6)		0.29	
	4	239 (26.8)	41 (19.3)		198 (29.1)		0.17	
	Missing	145 (16.3)	38 (17.9)		107 (15.7)		0.26	
Performance Status	0	483 (54.1)	109 (51.4)		374 (55.0)		0.23	0.001
	1	279 (31.3)	83 (39.2)		196 (28.8)		0.3	
	2	44 (4.9)	2 (0.9)		42 (6.2)		0.05	
	3	10 (1.1)	0 (0.0)		10 (1.5)		0	
	4	1 (0.1)	0 (0.0)		1 (0.1)		0	
	Missing	75 (8.4)	18 (8.5)		57 (8.4)		0.24	
IMD Decile	1st Tertile	276 (30.9)	45 (21.2)		231 (34.0)		0.16	0.004
	2nd Tertile	393 (44.1)	104 (49.1)		289 (42.5)		0.26	
	3rd Tertile	216 (24.2)	57 (26.9)		159 (23.4)		0.26	
	Missing	7 (0.8)	6 (2.8)		1 (0.2)		0.86	
Income Decile	1st Tertile	304 (34.1)	52 (24.5)		252 (37.1)		0.17	0.006
	2nd Tertile	385 (43.2)	99 (46.7)		286 (42.1)		0.26	
	3rd Tertile	196 (22.0)	55 (25.9)		141 (20.7)		0.28	
	Missing	7 (0.8)	6 (2.8)		1 (0.2)		0.86	

**Table 2:** Univariate analysis. Age is treated as a continuous variable, with the odds ratio reflecting the increasing likelihood of trial participation with every increasing year of age. The IMD tertile is treated as ordinal, and the remaining variables are treated as binary.

Variable	Odds Ratio	95% CI Lower	95% CI Upper	*p*-Value
Age	0.85	0.73	0.99	0.038
Ethnicity—White	1.39	1.18	1.64	<0.001
English-Speaking	1.23	1.02	1.49	0.032
Interpreter Required	0.72	0.58	0.91	0.005
Performance Status	0.92	0.78	1.08	0.312
IMD tertile	1.25	1.07	1.46	0.006

**Table 3:** Multivariate analysis. Age is treated as a continuous variable, with the odds ratio reflecting the increasing likelihood of trial participation with every increasing year of age, and the remaining variables are treated as binary.

Variable	Odds Ratio	95% CI Lower	95% CI Upper	*p*-Value
Age	0.79	0.67	0.93	0.003
IMD decile	1.36	1.09	1.69	0.007
Interpreter Required	0.4	0.17	0.95	0.037
Ethnicity—White	1.89	1.33	2.68	<0.001

## Data Availability

Data are available on reasonable request from the corresponding author, following approval from both involved NHS Trusts.

## Supplementary Materials

The supporting information can be downloaded at https://www.mdpi.com/article/10.3390/cancers16213590/s1.
