# Supplementary material for: Ethnicity and Socioeconomic Disparities in Clinical Trial Participation for Ovarian Cancer: A Retrospective Observational Study in London
Source: Cancers (Basel). 2024 Oct 24;16(21):3590. doi: 10.3390/cancers16213590 (PMC11545063; doi:10.3390/cancers16213590)
Supplement: Supplementary file 1 [file cancers-16-03590-s001.zip › cancers-3134190-supplementary.pdf]

Supplementary Analysis

Between-Hospital Differences

Patients at UCLH were generally younger than those treated at SBH ($p = 0.022$), and there were noticeable differences in the ethnic makeup of the cohorts from each hospital ($p = 0.003$). A larger proportion of patients at SBH were non-English speaking and significantly more required the assistance of a translator compared to UCLH ($p = 0.041$ and 0.005, respectively). Additionally, more patients at SBH were unemployed in comparison to those at UCLH ($p = 0.022$). Differences were also observed in the staging and performance status of the patients treated at both hospitals ($p = 0.001$). Socioeconomic factors such as the Index of Multiple Deprivation (IMD) and income tertiles also varied, with fewer patients at SBH falling into the higher income tertiles ($p = 0.004$ and $p = 0.006$, respectively).

Table S1. Age descriptives for both hospitals and the between-hospital differences, following a Mann-Whitney U test.

Feature	All Median (IQR)	Trial Median (IQR)	Non-Trial Median (IQR)	*p*-Value	Between-Hospital Diff. (*p*-Value)
SBH	62.0 (52.0 - 71.0)	59.0 (52.0 - 67.0)	64.0 (52.0 - 72.8)	0.005*	
UCLH	59.0 (47.5 - 70.5)	55.0 (46.0 - 64.0)	59.0 (48.0 - 71.8)	0.1	0.022*

Table S2. Categorical descriptives for both hospitals and the between-hospital differences, following a chi-squared test.

Feature	Sub-Group	UCLH					SBH					Between-Hospital Diff. (*p*-Value)
		**All (N = 617)**	**Trial (N = 163)**	**Non-Trial (N = 454)**	**Trial:All**	***p*-Value**	**All (N = 275)**	**Trial (N = 49)**	**Non-Trial (N = 226)**	**Trial:All**	***p*-Value**	
	Asian	61	13 (8.0%)	48 (10.6%)	0.21		48	4 (8.2%)	44 (19.5%)	0.08		
	Black	45	7 (4.3%)	38 (8.4%)	0.16		34	4 (8.2%)	30 (13.3%)	0.12		
	Other	33	8 (4.9%)	25 (5.5%)	0.24		17	2 (4.1%)	15 (6.6%)	0.12		
Ethnicity	White	376	119 (73.0%)	257 (56.6%)	0.32	0.061	169	35 (71.4%)	134 (59.3%)	0.21	0.155	0.003*
	No	41	4 (2.5%)	37 (8.1%)	0.1		28	5 (10.2%)	23 (10.2%)	0.18		
English Speaking?	Yes	575	159 (97.5%)	416 (91.6%)	0.28	0.02*	246	44 (89.8%)	202 (89.4%)	0.18	1	0.041*
	No	573	159 (97.5%)	414 (91.2%)	0.28		249	47 (95.9%)	202 (89.4%)	0.19		
Translator Required?	Yes	42	4 (2.5%)	38 (8.4%)	0.1	0.016*	25	2 (4.1%)	23 (10.2%)	0.08	0.281	0.005*
	No	44	6 (3.7%)	38 (8.4%)	0.14		26	2 (4.1%)	24 (10.6%)	0.08		
Employed	Yes	376	101 (62.0%)	275 (60.6%)	0.27	0.085	143	26 (53.1%)	117 (51.8%)	0.18	0.3	0.022*
	1	116	23 (14.1%)	93 (20.5%)	0.2		22	4 (8.2%)	18 (8.0%)	0.18		
	2	20	7 (4.3%)	13 (2.9%)	0.35		21	5 (10.2%)	16 (7.1%)	0.24		
	3	217	66 (40.5%)	151 (33.3%)	0.3		112	28 (57.1%)	84 (37.2%)	0.25		
Stage	4	150	32 (19.6%)	118 (26.0%)	0.21	0.07	89	9 (18.4%)	80 (35.4%)	0.1	0.056	0.007*
	0	353	85 (52.1%)	268 (59.0%)	0.24		130	24 (49.0%)	106 (46.9%)	0.18		
	1	181	72 (44.2%)	109 (24.0%)	0.4		98	11 (22.4%)	87 (38.5%)	0.11		
	2	23	2 (1.2%)	21 (4.6%)	0.09		21	0 (0.0%)	21 (9.3%)	0		
	3	6	0 (0.0%)	6 (1.3%)	0		4	0 (0.0%)	4 (1.8%)	0		
Performance Status	4	0	0 (0.0%)	0 (0.0%)	0	< 0.001**	1	0 (0.0%)	1 (0.4%)	0	0.131	0.001*
	0	146	27 (16.6%)	119 (26.2%)	0.18		130	18 (36.7%)	112 (49.6%)	0.14		
	1	284	83 (50.9%)	201 (44.3%)	0.29		109	21 (42.9%)	88 (38.9%)	0.19		
IMD tertile	2	180	47 (28.8%)	133 (29.3%)	0.26	0.054	36	10 (20.4%)	26 (11.5%)	0.28	0.136	0.004*
	1st Tertile	179	32 (19.6%)	147 (32.4%)	0.18		125	20 (40.8%)	105 (46.5%)	0.16		
	2nd Tertile	271	80 (49.1%)	191 (42.1%)	0.3		114	19 (38.8%)	95 (42.0%)	0.17		
Income tertile	3rd Tertile	160	45 (27.6%)	115 (25.3%)	0.28	0.016*	36	10 (20.4%)	26 (11.5%)	0.28	0.244	0.006*

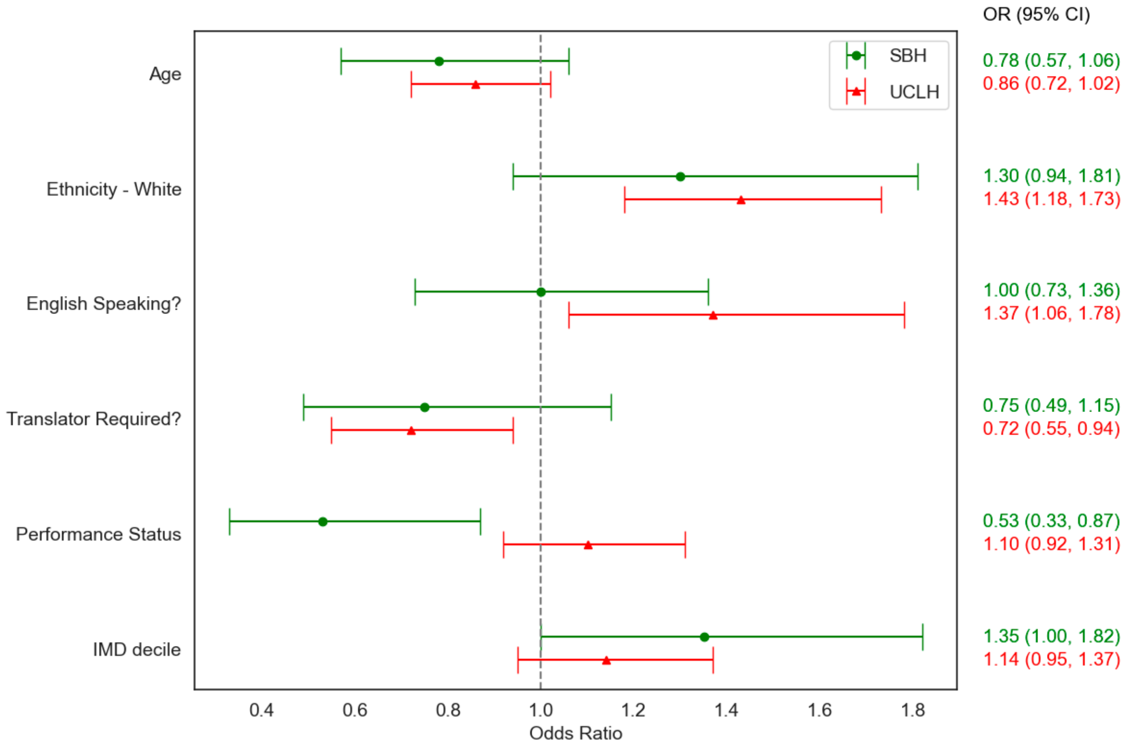

Figure S1. Forest plot demonstrating the independent associations of each predictor with trial participation for both hospitals. Age is treated as a continuous variable with the odds ratio reflecting the increasing likelihood of trial participation with every increasing year of age. IMD tertile is treated as ordinal and the remaining variables are treated as binary.

Machine Learning Approach to Determining Factors Affecting Trial Participation.

Methods

Model Development

The dataset was pre-processed by filling rows with missing values in the predictor using median imputation. An XGBoost classifier was trained using nested cross-validation for hyperparameter tuning. The outer cross-validation consisted of a stratified 5-fold split, while the inner cross-validation, used for hyperparameter optimization, employed a stratified 4-fold split. Hyperparameters were tuned using grid search within the inner cross-validation loop. The grid search explored the following parameter ranges:

- n_estimators: [50, 100, 200]
- max_depth: [3, 4, 5, 6]
- learning_rate: [0.01, 0.1, 0.2]
- subsample: [0.8, 0.9, 1.0]
- colsample_bytree: [0.8, 0.9, 1.0]
- scale_pos_weight: [1, ratio of negative to positive samples in the training set]

The best model was selected based on maximising the area under the receiver operating characteristic curve (AUROC).

Evaluation Metrics

Performance metrics were computed for each fold of the outer cross-validation. We calculated accuracy, sensitivity (recall), specificity, and AUROC. Confusion matrices were generated to calculate specificity, and model performance metrics were averaged across all folds to provide mean and standard deviation.

Model Interpretation

To interpret the model and understand how each feature impacted the likelihood of trial participation, SHAP (SHapley Additive exPlanations) values were computed using the TreeExplainer method for each fold's test set. SHAP values were concatenated across all folds, and a summary plot of feature importance was generated using the combined test data and SHAP values.

Results

The performance of the classifier in predicting which patients were going to participate in a trial was as follows:

- Sensitivity: 0.70 ± 0.10
- Specificity: 0.53 ± 0.05
- AUROC: 0.67 ± 0.05

Performance metrics demonstrate whilst trial performance can be indicated to some degree with the variables explored in this study, there are likely other factors to consider. Additionally, given the sample size, our results are subject to a degree of variance, evidenced by the large standard deviations.

Figure S2 presents a bee swarm plot demonstrating the feature importance of the model. Of all the explored features, age proved most predictive, with both older and younger ages causing the model to be less likely to predict a patient to be part of the trial cohort, suggesting the relationship between age and trial participation is not linear. The second most important variable was whether the patient was White, with White patients causing the model to increase the likelihood of predicting the patient as being a trial participant. If the patient was in the lowest tertile, the model was less likely to predict them as a trial participant, with little distinction made between the 2nd and 3rd tertile in terms of their influence on trial participation. Much like age, the relationship between trial participation and performance status was observed to be non-linear, with patients in stage 1 being most likely to be predicted as being a trial participant. The requirement for a translator caused the model to be less likely to predict the patient as a trial participant. English speaking was not found to significantly affect the model’s predictions for trial participation.

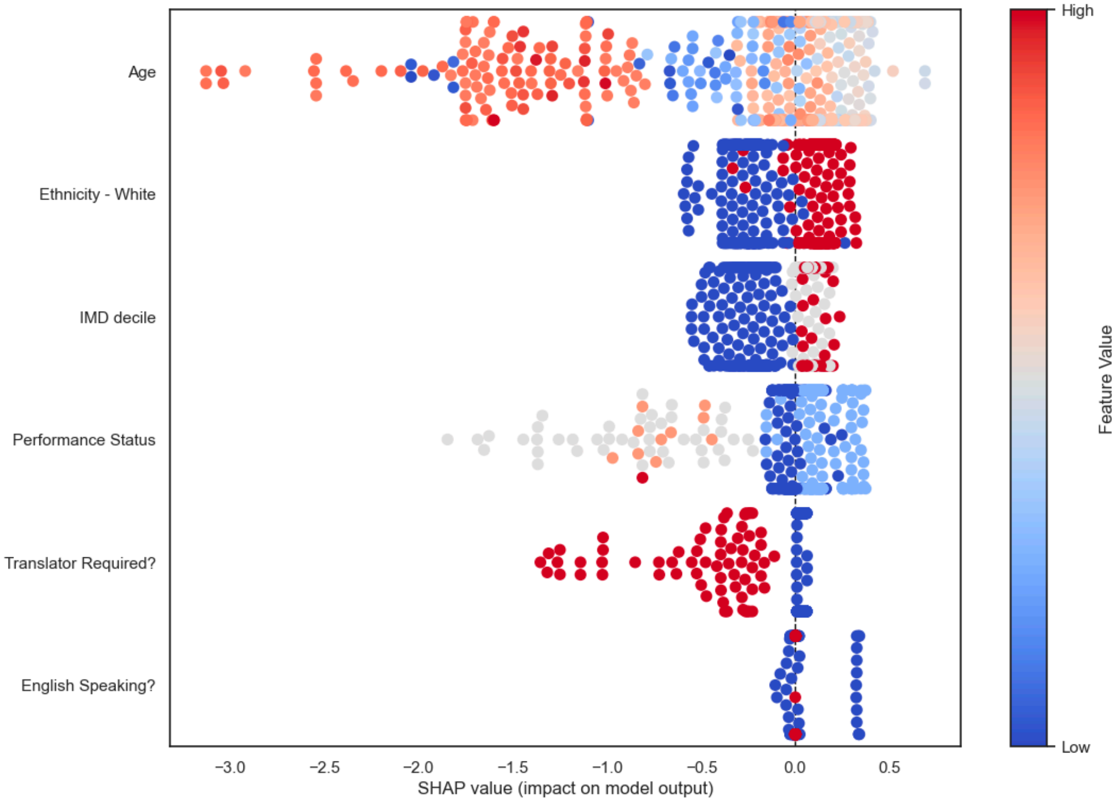

Figure S2. SHAP beeswarm plot illustrating feature importance for the XGBoost model. Each point represents a SHAP value for a single feature across different samples, with colour indicating the value of the feature (red for high values, blue for low values). Features are ranked vertically by their overall impact on the model's predictions, with the most important features at the top. The horizontal spread of the points shows how much each feature contributes (positively or negatively) to the prediction for each instance. Points further from zero indicate a larger influence on the model's decision.
